# Body composition impacts outcome of bronchoscopic lung volume reduction in patients with severe emphysema: a fully automated CT-based analysis

**DOI:** 10.1038/s41598-024-58628-0

**Published:** 2024-04-15

**Authors:** Johannes Wienker, Kaid Darwiche, Nele Rüsche, Erik Büscher, Rüdiger Karpf-Wissel, Jane Winantea, Filiz Özkan, Dirk Westhölter, Christian Taube, David Kersting, Hubertus Hautzel, Luca Salhöfer, René Hosch, Felix Nensa, Michael Forsting, Benedikt M. Schaarschmidt, Sebastian Zensen, Jens Theysohn, Lale Umutlu, Johannes Haubold, Marcel Opitz

**Affiliations:** 1 Division of Interventional Pneumology, Department of Pulmonary Medicine, University Medicine Essen-Ruhrlandklinik, Tüschener Weg 40, 45239 Essen, Germany; 2 Department of Pulmonary Medicine, University Medicine Essen-Ruhrlandklinik, Essen, Germany; 3 Institute for Artificial Intelligence in Medicine, University Hospital Essen, Essen, Germany; 4 Institute of Diagnostic and Interventional Radiology and Neuroradiology, University Hospital Essen, Essen, Germany; 5 Department of Nuclear Medicine, University Hospital Essen, Essen, Germany

**Keywords:** Chronic obstructive pulmonary disease, Emphysema, Bronchoscopic lung volume reduction, Valves, Body composition, Computed tomography, Deep learning, Artificial intelligence, Chronic obstructive pulmonary disease, Three-dimensional imaging, Radiography

## Abstract

Chronic Obstructive Pulmonary Disease (COPD) is characterized by progressive and irreversible airflow limitation, with individual body composition influencing disease severity. Severe emphysema worsens symptoms through hyperinflation, which can be relieved by bronchoscopic lung volume reduction (BLVR). To investigate how body composition, assessed through CT scans, impacts outcomes in emphysema patients undergoing BLVR. Fully automated CT-based body composition analysis (BCA) was performed in patients with end-stage emphysema receiving BLVR with valves. Post-interventional muscle and adipose tissues were quantified, body size-adjusted, and compared to baseline parameters. Between January 2015 and December 2022, 300 patients with severe emphysema underwent endobronchial valve treatment. Significant improvements were seen in outcome parameters, which were defined as changes in pulmonary function, physical performance, and quality of life (QoL) post-treatment. Muscle volume remained stable (1.632 vs. 1.635 for muscle bone adjusted ratio (BAR) at baseline and after 6 months respectively), while bone adjusted adipose tissue volumes, especially total and pericardial adipose tissue, showed significant increase (2.86 vs. 3.00 and 0.16 vs. 0.17, respectively). Moderate to strong correlations between bone adjusted muscle volume and weaker correlations between adipose tissue volumes and outcome parameters (pulmonary function, QoL and physical performance) were observed. Particularly after 6-month, bone adjusted muscle volume changes positively corresponded to improved outcomes (ΔForced expiratory volume in 1 s [FEV_1_], r = 0.440; ΔInspiratory vital capacity [IVC], r = 0.397; Δ6Minute walking distance [6MWD], r = 0.509 and ΔCOPD assessment test [CAT], r = −0.324; all *p* < 0.001). Group stratification by bone adjusted muscle volume changes revealed that groups with substantial muscle gain experienced a greater clinical benefit in pulmonary function improvements, QoL and physical performance (ΔFEV_1_%, 5.5 vs. 39.5; ΔIVC%, 4.3 vs. 28.4; Δ6MWDm, 14 vs. 110; ΔCATpts, −2 vs. −3.5 for groups with ΔMuscle, BAR% < –10 vs. > 10, respectively). BCA results among patients divided by the minimal clinically important difference for forced expiratory volume of the first second (FEV_1_) showed significant differences in bone-adjusted muscle and intramuscular adipose tissue (IMAT) volumes and their respective changes after 6 months (ΔMuscle, BAR% −5 vs. 3.4 and ΔIMAT, BAR% −0.62 vs. 0.60 for groups with ΔFEV1 ≤ 100 mL vs > 100 mL). Altered body composition, especially increased muscle volume, is associated with functional improvements in BLVR-treated patients.

## Introduction

Chronic Obstructive Pulmonary Disease (COPD) represents a progressive systemic disorder with considerable implications for patient health. Prolonged exposure to inhaled noxious agents, predominantly inhalation of tobacco, elicits chronic inflammation in the small airways culminating in airway obstruction and consequential damage to lung parenchyma. As the condition advances, there is a gradual loss of parenchymal tissue and diminished elastic recoil, thus facilitating the emergence of pulmonary emphysema. At all stages, patients experience substantial declines in lung function, quality of life and exercise capacity^1^.

COPD triggers systemic inflammation, impacting body composition and leading to a sedentary lifestyle, reduced muscle mass, and heightened mortality risk. Physical activity level strongly predicts overall mortality in COPD patients. Skeletal muscle mass notably influences pulmonary function, with studies linking sarcopenia and decreased muscle mass to impaired parameters. Skeletal muscle mass notably influences pulmonary function, with studies linking sarcopenia and decreased muscle mass to impaired parameters^2–7^.

Smoking cessation is crucial and early relief can come from physical conditioning and medical intervention. Advanced stages may require invasive approaches, like bronchoscopic lung volume reduction, improving pulmonary function, exercise capacity and QoL as evidenced in several randomized clinical trials, particularly for cases with compromised ventilatory mechanics^7–11^.

To date, various techniques have been assessed for the evaluation of body composition in patients with COPD. These include, among others, manual CT-derived muscle morphometry, muscle strength measurements, and bioelectrical impedance analysis (BIA)^12–15^. However, no universally accepted gold standard has been established for monitoring body composition in COPD patients, as each method comes with its own set of advantages and limitations.

Recent advancements in deep learning have given rise to novel approaches in CT-based body composition analysis (BCA), employing a multi-resolution 3D U-Net for feature extraction^16–18^. These techniques facilitate fully automated segmentation of volumetric body composition data obtained from CT scans, replacing the need for manual or semi-automatic segmentation methods. In this study, we utilized a fully automated CT-based 3D BCA approach.

Given these considerations, our study seeks to investigate the potential significance of fully automated CT-based body composition analysis in elucidating the outcome of endoscopic lung volume reduction in COPD patients. This investigation specifically focuses on the identification of pivotal parameters within body composition and their corresponding alterations, aiming to discern their integral role in the procedural success.

To our knowledge, this represents the first comprehensive examination of the potential influence of body composition on the outcome after this intervention. By addressing this knowledge gap, our research endeavors to contribute valuable insights that could potentially enhance the precision and efficacy of treatment strategies for COPD patients, ultimately leading to improved clinical outcomes and a higher quality of life for individuals suffering from this debilitating condition.

## Methods

### Study population

In this retrospective single-center trial, we conducted an evaluation of patients suffering from end-stage emphysema, who received treatment with one-way endobronchial valves (Zephyr© Endobronchial Valve; Pulmonx, Inc., Redwood City, CA, USA) between January 2015 and December 2022. For the comprehensive assessment of body composition before and after valve placement, only patients who underwent pre-interventional high-resolution computed tomographies (HR-CT) for emphysema quantification were included in the study.

Patients were considered as possible candidates for bronchoscopic lung volume reduction (BLVR) with valves after exhausting medical and supportive interventions, and a forced expiratory volume in 1 s (FEV_1_) of less than 40% predicted and a residual volume (RV) exceeding 200% predicted. Prior to valve implantation, each patient underwent a standardized diagnostic protocol to ensure optimal conditions. The assessment of collateral ventilation was carried out through QCT analysis to evaluate fissure completeness (StratX^©^ software, Pulmonx, Inc.), in conjunction with the use of the Chartis catheter-based system (Pulmonx, Inc.).

In addition to pulmonary function tests, our evaluation included a perfusion scintigraphy, echocardiography to rule out cardiac insufficiency, and a 6-min walking test (6MWT) to assess physical fitness. The health-related quality of life was gauged using the COPD Assessment Test (CAT).

All cases were subjected to thorough discussion within a multidisciplinary board, comprising interventional pneumologists, thoracic surgeons, and radiologists, in order to arrive at the optimal treatment decisions. Preceding BLVR, all patients at our center underwent pulmonary rehabilitation and refrained from smoking for a minimum of 6 months, which was verified through normal serum carboxyhemoglobin (COHb) and urinary cotinine levels.

Following valve placement, patients were closely monitored for 24 h and not discharged within four days after valve placement to ensure prompt responsiveness in the event of a pneumothorax. Initial follow-up assessments were scheduled at 3- and 6-months post-implantation, encompassing repeat pulmonary function tests, the 6MWT and the CAT.

High-resolution computed tomography (HR-CT) scans were also performed at the 3- and 6-month follow-up points to quantify post-interventional emphysema changes, assess the extent of volume reduction, and verify the proper function and placement of the implanted valves. By combining these results, one can objectively evaluate the overall effectiveness of the treatment.

The study was conducted in compliance with the guidelines of the Institutional Review Board of the University Hospital Essen. The Ethics Committee of the University of Duisburg-Essen (Approval Number 22-11074-BO) waived the informed consent due to the retrospective and anonymous nature of this study. The data were completely anonymized before being included in the study.

### CT acquisition

High-resolution non-contrast-enhanced chest CT scans were performed using a 64-detector row single-source computed tomography scanner (SOMATOM Definition AS, Siemens Medical Solutions, Forchheim, Germany). The scanner had a gantry rotation time of 300 ms, with collimation set at 64 × 0.6 mm. The tube current–time product per rotation was 110 mAs, and the tube voltage was set to 120 kV. Image reconstruction utilized convolution kernels B31F. To minimize radiation exposure, we employed the CARE Dose4D and CARE kV algorithms (Siemens Medical Solutions, Forchheim, Germany). Patients were scanned in a head-first supine position with their arms elevated during an inspiratory breath-hold. All scans were resampled to a slice thickness of 1.0 mm for utilization in the body composition network.

### Body composition analysis

Automated BCA at baseline was performed using chest CT scans obtained from all participants in the study and body composition characteristics extracted from these chest CT scans were obtained using a pre-trained deep learning network, which represents a progression of the system detailed by Koitka et al.^16^. An open-source body and organ analysis (BOA) tool is accessible for universal utilization. This availability enhances the transparency and reproducibility of the examined method^19^. This network utilizes a variant of the multi-resolution 3D U-Net architecture and enables the fully automated segmentation of tissues within identified body regions in CT scans^18^. Within the scope of this research, the Body Composition Analysis (BCA) assessed the volume (in milliliters) of four distinct adipose tissue markers: subcutaneous adipose tissue (SAT), intra- and intermuscular adipose tissue (IMAT), epicardial adipose tissue (EAT), and paracardial adipose tissue (PAT).

**Figure 1:**
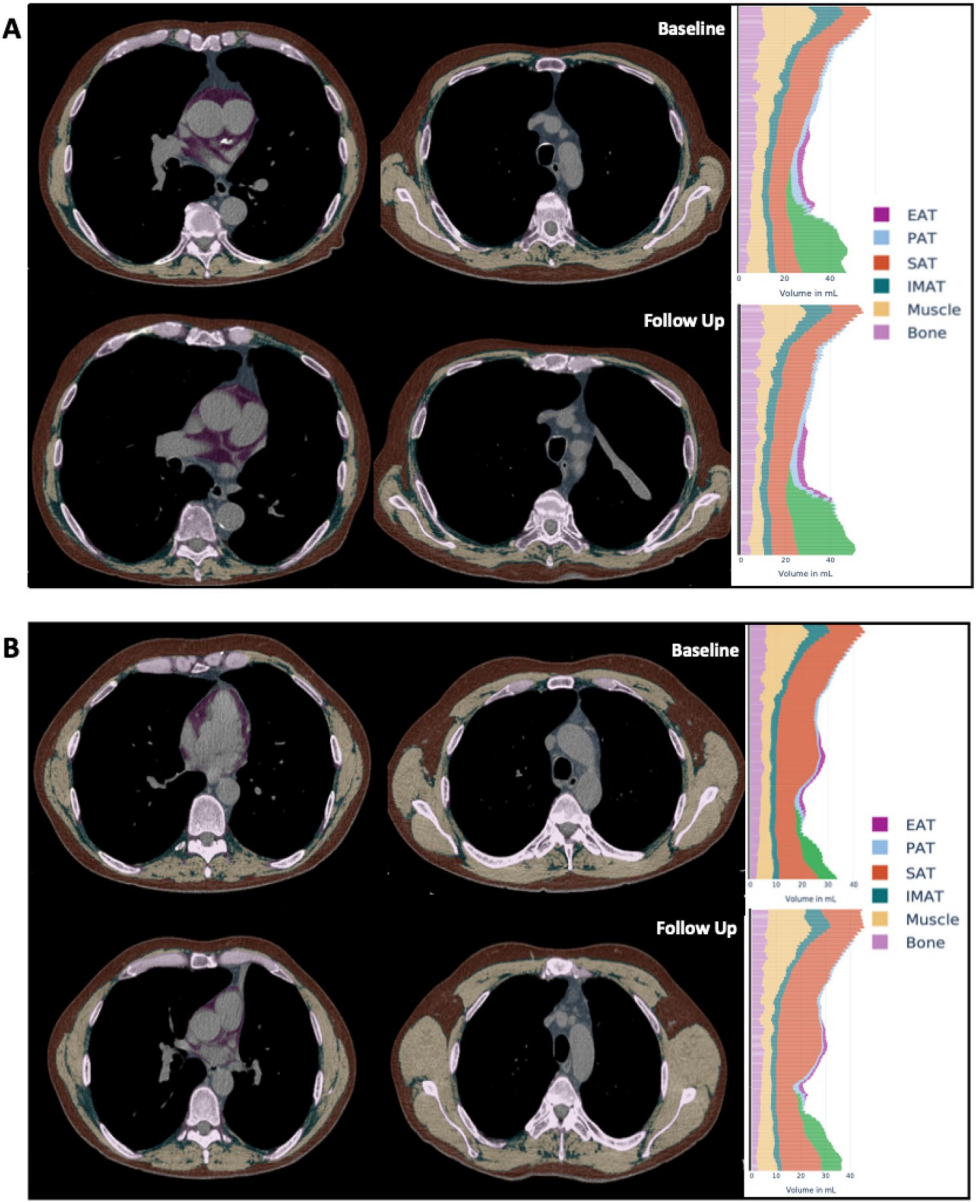
Exemplary outputs of a fully automated CT-based body composition analysis (BCA) before and after BLVR. The BCA network detects various BCA features within the chest CT scan, which are combined with bone volume to calculate body size-adjusted biomarkers. Individual volume distribution is graphically presented. Tissues are marked by colors: pink for bone, yellow for muscle, orange-brown for subcutaneous adipose tissue (SAT), purple for epicardial adipose tissue (EAT), blue for paracardial adipose tissue (PAT), and turquoise for inter- and intramuscular adipose tissue (IMAT). Shown here is an illustrative analysis comparing a patient with a decrease in muscle volume (**A**) to another patient with an increase in muscle volume (**B**). Visceral adipose tissue (VAT), represented in green, was not included in this analysis due to variations in the abdominal area covered by the scans among patients.

Muscular tissue and bone volume were quantified based on the generated segmentations. The raw BCA features underwent normalization by bone volume to yield body size-adjusted ratios (BARs) for muscle and adipose tissue measurements (Fig. 1). Normalization using bone volume enables a scan-specific adjustment, even with varying scan areas across different examinations, a condition not achievable through standardization based on a constant body size.

### Outcome variables

The fully automated BCA reports provided detailed information regarding individual bone volume, muscle mass, and various types of adipose tissue volumes, along with their corresponding interrelated proportions. We conducted a comparative analysis between the BCA results and baseline parameters, as well as the outcomes following Bronchoscopic Lung Volume Reduction (BLVR). These outcomes were determined by changes in pulmonary function metrics (FEV_1_, RV, and IVC), physical performance (6MWT), and quality of life (CAT).

Pulmonary function assessments were performed using body plethysmography and spirometry according to ERS/ATS guidelines, while the 6-min walk test (6MWT) adhered to standard protocols according to ATS guidelines and included data on oxygen saturation, the use of walking aids, and supplemental oxygen requirements^20,21^. CAT score was determined based on a standardized questionnaire.

### Statistical analysis

The Parameters were presented as mean and standard deviation or median with range, as indicated. To assess changes in outcome parameters from baseline after 3 and 6 months, we conducted paired-samples t-tests for data with a parametric distribution and Wilcoxon signed-rank tests for data with a non-normal distribution.

To explore the relationship between BCA results and outcome parameters, as well as their changes at baseline and post-implantation during the 3- and 6-month follow-ups, we initially conducted a non-parametric Spearman correlation analysis (r_s_). The most impactful parameter identified was the BAR of muscle volume, which was then used to establish four *a-priori* defined groups (relative changes in muscle, BAR < –10%, ≤ 0%, > 0% and > 10%) for further investigation of the interplay between BCA and functional baseline and outcome parameters.

Furthermore, we examined the relationship from the opposite perspective by categorizing the study population into those who achieved the accepted minimal clinically important difference (MCID) for the pulmonary function parameter FEV_1_ at the 6-month mark (+ 100 mL) after valve implantation, enabling inter-group comparisons of BCA results^22^. Two-Group differences were assessed using an independent-samples t-test in case of parametric distribution or Mann–Whitney-Test in case of non-parametric distribution. Four-group differences were analyzed using one-way analysis of variance (ANOVA) for data with parametric data and Kruskal–Wallis ANOVA for data with non-parametric data. The aforementioned analysis was again repeated for those who achieved the MCID (−310 mL) for residual volume (RV) and those who did not (Supplementary Material)^23^. Differences in categorical data were assessed using a chi-squared test. In case of multiple comparisons, Bonferroni-adjusted *p*-values were used.

Variables were subjected to the Shapiro–Wilk Test to determine non-normal distribution. A *p*-value of less than 0.05 was considered statistically significant. All statistical analyses and graph preparations were conducted using SPSS version 23 (IBM, New York, NY, USA).

### Approval for human experiments

The experiments of the study were approved by the Ethics Committee of the University of Duisburg-Essen (approval number approval number 22-11074-BO). The experiments were performed in accordance with the declaration of Helsinki and with all guidelines set forth by the approving Institutional Ethics Committee. The data were completely anonymized before being included in the study.

## Results

### Study population and procedural results

**Figure 2:**
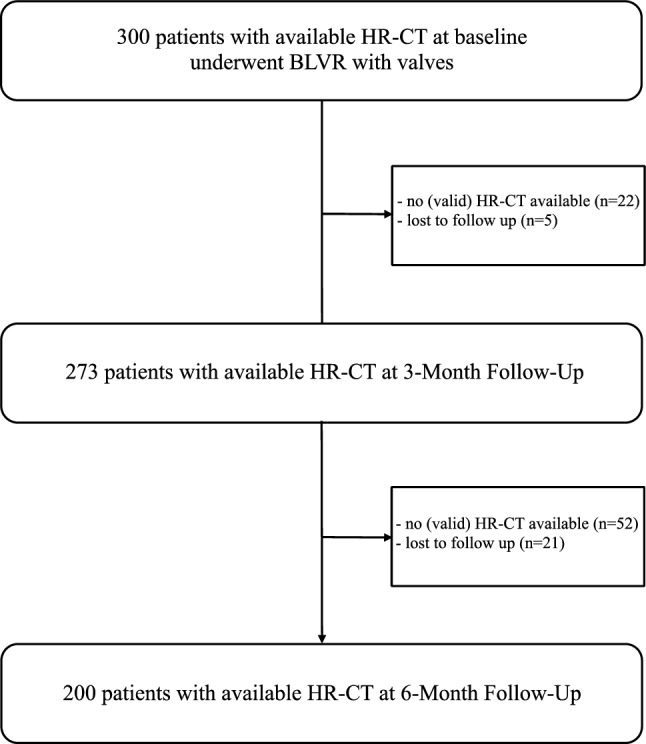
Data availability flow chart. HR-CT, high resolution computed tomography; BLVR, bronchoscopic lung volume reduction.

Between January 2015 and December 2022, we identified a total of 300 patients with a median age of 58 years and a range of 38–82 years who underwent endobronchial valve treatment at our center and had HR-CT scans at baseline. Three months after the intervention, follow-up HR-CT scans were available for 273 patients. At the 6-month follow-up, HR-CT scans were accessible for 200 patients. In 79 cases, either no HR-CT scan was performed or the available scan was not valid at either the 3-month (n = 22) or 6-month (n = 52) post-intervention time points. Notably, there were inconsistencies in CT data collection for body composition analysis in 14 out of these 79 cases. Additionally, at the 3-month mark, 5 patients, and at the 6-month mark, 21 patients were lost to follow-up or had their follow-up appointments postponed. Detailed baseline demographic, clinical and body composition data can be found in Table 1. A data availability flow chart is displayed in Fig. 2.

**Table 1:** Demographics, clinical characteristics and body composition at baseline.

Parameter	Median (range) or number (no.)
Female/male, n (%)	181/119 (60/40)
Age, years	58 (38–82)
Weight, kg	65 (38–125)
Height, cm	170 (148–197)
Body mass index, kg/m^2^	22.6 (14–37)
Smoking history, pack-years	40 (0–150)
FEV_1_	
Liters	0.75 (0.3–1.6)
% predicted	27 (8–47)
RV	
Liters	5.7 (3.2–11.6)
% predicted	263 (163–474)
IVC	
Liters	2.2 (0.6–4.6)
% predicted	67 (13–112)
CAT score (n = 215)	27 (14–38)
6MWD, meters	280 (95–520)
Bone, volume (mL)	1433 (970–2484)
Muscle	
Volume (mL)	2384 (1290–4973)
Bone adjusted ratio (BAR)	1.63 (0.9–2.7)
Total adipose tissue (TAT)	
Volume (mL)	4532 (338–14,694)
Bone adjusted ratio (BAR)	2.86 (0.25–10.3)
SAT, BAR	1.95 (0.08–7.9)
PAT, BAR	0.16 (0.03–0.58)
IMAT, BAR	0.54 (0.09–1.87)
EAT, BAR	0.08 (0.01–0.28)

Values are median (range) or number (no.)

*6MWD* 6-min walking distance, *CAT* COPD assessment test, *FEV*_*1*_ forced expiratory volume in 1 s, *IVC* inspiratory vital capacity, *RV* residual volume, *BAR* bone adjusted ratio, *EAT* epicardial adipose tissue, *PAT* pericardial adipose tissue, *IMAT* intramuscular adipose tissue, *SAT* subcutaneous adipose tissue, *TAT* total adipose tissue.

All patients received treatment with Zephyr endobronchial valves, and the occlusion of the treated lobe was confirmed by bronchoscopy immediately after placement as well as CT scans and bronchoscopy after 3 and 6 months. In the majority of cases, the left lung was treated, with the left upper lobe (LUL) being the most frequently targeted (left lung n = 207 [69%]; LUL, n = 115 and left lower lobe (LLL), n = 92). In contrast, in the right lung, the lower lobe (RLL) was more commonly targeted (right lung n = 93 [31%]; right upper lobe (RUL), n = 43; right middle lobe (RML), n = 5; RLL, n = 45).

At the 3 and 6-month follow-up assessments, patients exhibited notable improvements in pulmonary function (FEV_1_ changed from 0.75 to 0.81L, RV from 5.7 to 4.9L and IVC from 2.2 to 2.5L), physical performance (6MWD improved from 280 to 310 m), and QoL (CAT improved from 27 to 25 points), indicating an overall advantage gained from BLVR treatment (Table 2).

**Table 2:** Changes in clinical parameters and body composition.

Parameter	Pre-implant (Baseline)	Follow-up (3-Month)	Follow-up (6-month)
	n = 300	n = 273	n = 200
FEV_1_, L	0.75 (0.3–1.6)	0.85 (0.38–1.9)***	0.81 (0.32–1.65)***
RV, L	5.7 (3.2–11.6)	4.9 (2.9–12.3)***	4.9 (3.0–9.5)***
IVC, L	2.2 (0.6–4.6)	2.56 (1.1–4.9)***	2.5 (0.86–4.9)***
6MWD, m	280 (95–520)	320 (110–545)***	310 (90–600)***
CAT score	27 (14–38)	24 (15–35)***	25 (12–37)***
Muscle, BAR	1.632 (0.9–2.7)	1.638 (1–3)	1.635 (0.77–3.2)
Δ, %	–	0.37	0.18
TAT, BAR	2.86 (0.25–10.3)	3.01 (0.34–14.7)*	3.00 (0.31–9)*
Δ, %	–	5.2	4.9
SAT, BAR	1.95 (0.08–7.9)	1.94 (0.1–6.1)	1.88 (0.09–6.4)
Δ, %	–	–0.51	–3.59
PAT, BAR	0.16 (0.03–0.58)	0.17 (0.04–0.53)	0.17 (0.04–0.5)*
Δ, %	–	6.3	6.3
IMAT, BAR	0.54 (0.09–1.87)	0.58 (0.1–2.74)	0.57 (0.12–1.44)
Δ, %	–	7.4	5.5
EAT, BAR	0.08 (0.01–0.28)	0.08 (0.02–0.3)	0.08 (0.02–0.28)
Δ, %	–	0	0

*6MWD* 6-min walking distance, *CAT* COPD assessment test, *FEV*_*1*_ forced expiratory volume in 1 s, *IVC* inspiratory vital capacity, *RV* residual volume, *BAR* bone adjusted ratio, *IMAT* intra- and intermuscular adipose tissue, *EAT* epicardial adipose tissue, *PAT* paracardial adipose tissue, *SAT* subcutaneous adipose tissue, *TAT* total adipose tissue. Statistically significant differences to baseline marked: *p < 0.05 **p < 0.01 ***p < 0.001.

In terms of patient’s safety, a pneumothorax occurred in 49 cases (16.4%), while a mild exacerbation was recorded in 19 patients (6.3%). Chest tube placement was necessary in 32 cases (32/49 = 65%).

### Body composition: baseline

The CT scans were performed at a median of 64 days prior to valve implantation, serving the dual purpose of emphysema quantification and fissure analysis.

At baseline, bone-adjusted muscle volume moderately correlated with FEV_1_, IVC, and 6MWD (rs = 0.491, 0.434, and 0.401, respectively; *p* < 0.001) but showed no significant correlation with RV. Regarding adipose tissues, there were weak negative correlations between TAT and SAT with RV and IVC ([RV] r_s_ = −0.207, −0.281 and [IVC] r_s_ = −0.194, −0.240, respectively; *p* < 0.001). IMAT exhibited weak negative correlations with IVC (r_s_ = −0.184, *p* < 0.001). Notably, the CAT score did not show any significant correlation with any of the BCA results.

### Body composition: follow-up

At the follow-up screenings, there was no statistically significant change in bone adjusted muscle volume. Regarding bone adjusted adipose tissue volumes, there was a significant change in TAT (BAR + 0.15, *p* < 0.05) at the 3-month follow-up mark. After 6-month there was a significant increase in TAT and PAT (BAR + 0.14 and + 0.01, respectively; *p* < 0.05) (Table 2).

In the correlation analysis, after the 3-month follow-up, bone-adjusted muscle volume exhibited a consistent moderate correlation with FEV_1_, IVC, and 6MWD, while a weak negative correlation was observed with the CAT score (r_s_ = 0.450, 0.444, 0.449, and −0.200, respectively; *p* < 0.001). Among bone adjusted adipose tissues, there were weak negative correlations between RV and TAT, SAT and IMAT (r_s_ = −0.309, −0.358 and −0.193, respectively; *p* < 0.001).

At the 6-month post-implantation mark, there was an increase in the correlation coefficients of bone-adjusted muscle volume, with a now strong correlation with FEV_1_ and 6MWD, as well as an increased moderate correlation with IVC and the CAT score (r_s_ = 0.610, 0.600, 0.564, and −0.506, respectively; *p* < 0.001). TAT, SAT, and IMAT displayed weak negative correlations with RV (r_s_ = −0.216, −0.269, and −0.146, respectively; *p* < 0.001).

**Figure 3:**
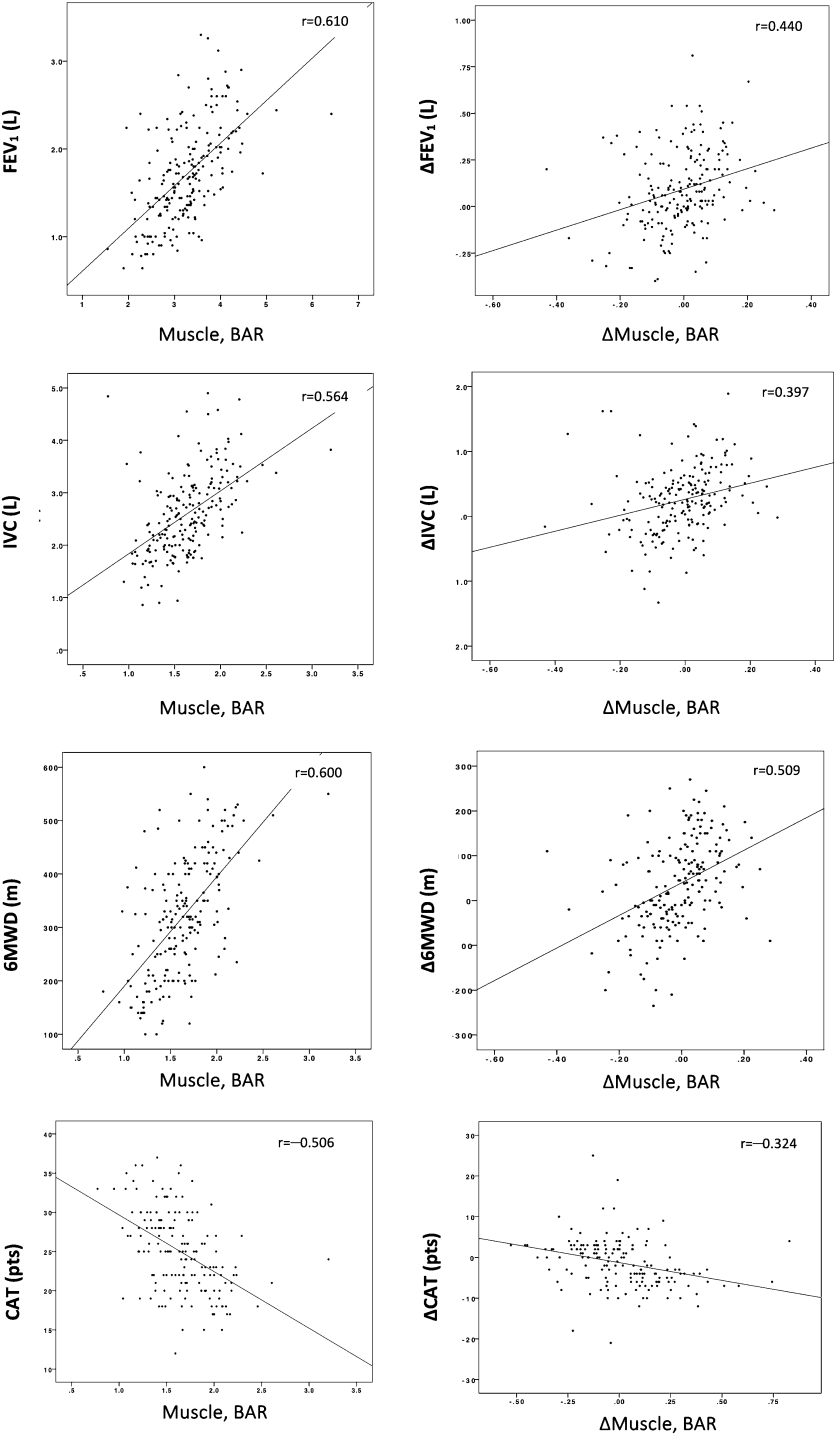
Correlation analysis of clinical outcomes with body composition parameters. Representative scatter plots of clinical outcome parameters with bone adjusted muscle volume (left) and their respective changes (right) at the 6-month follow-up mark. 6MWD, 6-min walking distance; CAT, COPD Assessment Test; FEV1, forced expiratory volume in 1 s; IVC, inspiratory vital capacity; RV, residual volume, BAR; bone adjusted ratio.

Furthermore, we examined the correlation of changes in bone-adjusted muscle volume and changes in TAT with changes in pulmonary function, 6MWD and the CAT score. After the 3-month follow-up, there was an initial weak correlation between changes in bone-adjusted muscle volume and changes in FEV_1_, IVC, 6MWD, and the CAT score. At the 6-month mark, the correlation coefficients between muscle volume and FEV_1_, IVC, and 6MWD increased to moderate levels, while the correlation with the CAT score became notably stronger (r_s_ = 0.201, 0.156, 0.263, and −0.01 versus r_s_ = 0.440, 0.397, 0.509, and −0.324, respectively; *p* < 0.001). No significant correlation was found for changes in TAT. For a comprehensive overview, correlation matrices for all time events and scatter plots depicting the correlation of parameters and their respective changes at the 6-month mark are displayed in Fig. 3 and supplementary figures S1 and S2.

A multiple linear regression analysis, adjusting for gender and age, was performed to evaluate the impact of body composition parameters and their respective changes on outcome parameters. All baseline body composition features were excluded from the final model due to their lack of statistically significant influence on the change in all outcome parameters (data not shown). The change in RV was subsequently incorporated into the final model to investigate the influence of volume reduction. In conjunction with the corresponding baseline parameter, the change in bone-adjusted muscle volume exhibited a statistically significant impact on all outcome parameters, while the volume reduction (ΔRV) demonstrated a significant impact on FEV1 and IVC (Table 3).

**Table 3:** Multivariate analysis for predictors of change of outcome parameters at the 6-month follow-up corrected for gender and age.

Parameter	Independents	B (95% CI)	*p*
ΔFEV_1_, mL	ΔRV	−0.44 (−.072 to −.017)	0.002
	FEV_1_ baseline	−0.262 (−.386 to −.139	< 0.001
	ΔMuscle, Bar	0.409 (.293 to .525)	< 0.001
ΔIVC, mL	ΔRV	−0.198 (−.262 to −.134)	< 0.001
	IVC baseline	−0.273 (−.375 to −.171)	< 0.001
	ΔMuscle, Bar	0.899 (.620 to 1.177)	< 0.001
Δ6MWD, m	ΔRV	−6.91 (−19.7 to 5.9)	0.288
	6MWD baseline	−0.347 (−.470 to −.223)	< 0.001
	ΔMuscle, Bar	226.2 (172 to 279)	< 0.001
ΔCAT, points	ΔRV	0.400 (−.386 to 1.19)	0.316
	CAT baseline	−0.457 (−.622 to −.292)	< 0.001
	ΔMuscle, Bar	−8.48 (−11 to −5)	< 0.001

*6MWD* 6-min walking distance, *CAT* COPD Assessment Test, *CI* confidence interval, *FEV*_*1*_ forced expiratory volume in 1 s, *IVC* inspiratory vital capacity, *RV* residual volume, *BAR* bone adjusted ratio.

### Group analysis

To explore the impact of muscle volume on outcome parameters, we conducted group differentiation based on muscle volume changes, resulting in four predefined groups. After the 3-month period, all groups showed improvements in outcome parameters, with the group experiencing a loss of muscle volume of more than 10 percent demonstrating statistically significant improvements in RV and CAT. Significant differences were observed in FEV_1_ and 6MWD, as well as their respective changes between the groups, although the difference doesn't follow a gradual increase pattern that corresponds directly to changes in muscle volume.

Conversely, for RV, IVC, and CAT score, along with their respective changes (except for changes in IVC), there are no discernible differences between the groups.

**Figure 4:**
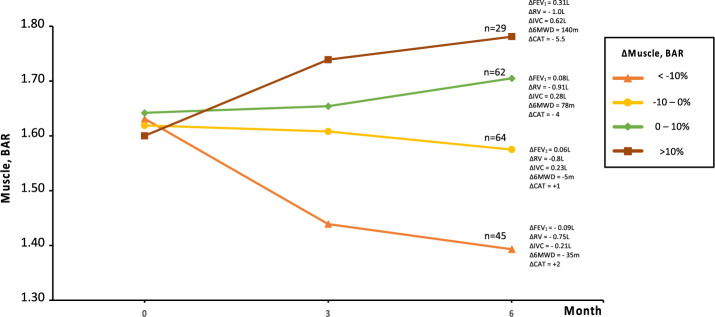
Graphical representation of changes in bone adjusted muscle volume (ΔMuscle, BAR at t = 6-month) along with the respective clinical outcome. 6MWD, 6-min walking distance; CAT, COPD Assessment Test; FEV1, forced expiratory volume in 1 s; IVC, inspiratory vital capacity; RV, residual volume, BAR; bone adjusted ratio.

At the 6-month follow-up, there is a significant difference between the groups for all outcome parameters and their respective changes, except for RV. Notably, there is now a gradual increase that corresponds to changes in muscle volume. After 6 months, the group with the highest degree of muscle loss exhibits outcome parameters that are worse than their baseline values. Table 4 shows the detailed values while Fig. 4 gives a graphically representation. The differences in clinical profile are displayed in supplementary table S3.

**Table 4:** Group differentiated analysis of outcome parameters according to changes in the bone adjusted ratio of muscle volume.

Parameter	Pre-implant (Baseline)	Follow-up (3-Month)					Follow-up (6-Month)				
ΔMuscle, BAR %		< -10	≤ 0	> 0	**> 10**		< -10	≤ 0	> 0	> 10	
*n*	300	32	113	98	30		45	64	62	29	
						*p*					*p*
FEV_1_, L	.75	.75	.80*	.99*	.92*	< *0.001*	.68*	.84*	.89*	.96*	< *0.001*
RV, L	5.7	5.1*	4.8*	4.9*	4.4*	***0.076***	4.9*	4.6*	4.8*	4.3*	***0.216***
IVC, L	2.2	2.3	2.3*	2.7*	2.7*	***0.293***	2.2	2.4*	2.9*	2.7*	< *0.001*
6MWD, m	280	260	295*	350*	365*	< *0.001*	250*	277	400*	395*	< *0.001*
CAT score	27	24*	24*	22*	24*	*0.473*	28*	27	22*	22*	< *0.001*
ΔFEV_1_, L		.03	.12	.10	.26	< *0.001*	–.09	.06	.08	.31	< *0.001*
ΔFEV_1_, %		5.5	17.9	11.5	39.5	< *0.001*	–11	9	10.5	43	< *0.001*
ΔRV, L		–.8	–.82	–.8	–.85	***0.509***	–.75	–.8	–.9	–1	***0.904***
ΔRV, %		–15	–16	–13	–17	***0.198***	–15	–16	–16	–19	***0.778***
ΔIVC, L		.09	.35	.23	.55	< *0.001*	–.21	.23	.28	.62	< *0.001*
ΔIVC, %		4.3	16.6	10	28.4	< *0.001*	–8.7	7.9	11	28.8	< *0.001*
Δ6MWD, m		14	25	54	110	< *0.001*	–35	–5	78	140	< *0.001*
ΔCAT score		–2	–2	–2	–3.5	***0.091***	2	1	–4	–5.5	< *0.001*

Significant values are in bolditalics, italics.

*6MWD* 6-min walking distance, *CAT* COPD assessment test, *FEV*_*1*_ forced expiratory volume in 1 s, *IVC* inspiratory vital capacity, *RV* residual volume, *BAR* bone adjusted ratio, *IMAT* intra- and intermuscular adipose tissue, *EAT* epicardial adipose tissue, *PAT* paracardial adipose tissue, *SAT* subcutaneous adipose tissue, *TAT* total adipose tissue. Values are displayed as median. Statistically significant differences between groups indicated with p-value and individual differences to baseline marked: *p < 0.001. We adjusted for multiple comparisons with Bonferroni correction.

When comparing the BCA results among patients divided by the FEV_1_-cut-off of 100 mL, which represents MCID, after the 6-month post-intervention period, statistically significant differences were observed only for bone-adjusted muscle and IMAT volumes, as well as their respective (relative) changes (Table 5).

**Table 5:** Group differentiated analysis of BCA parameters stratified by MCID for FEV_1_.

Parameter	Pre-implant (baseline)	Follow-Up (6-month)		*p*-value
ΔFEV_1_, mL		≤ 100	> 100	
		n = 114	n = 86	
Muscle, BAR	1.63 (0.9–3.2)	1.53 (0.8–3.2)	1.72 (1.0–2.6)	< 0.01
ΔMuscle, BAR	-	–0.07	0.068	< 0.01
ΔMuscle, BAR%	-	–5	3.4	< 0.01
TAT, BAR	2.86 (0.25–10.3)	3.16 (0.3–10.3)	3.6 (1.0–2.6)	n.s
ΔTAT, BAR	-	0.12	0.13	n.s
ΔTAT, BAR%	-	4	3.7	n.s
SAT, BAR	1.94 (0.8–7.9)	1.8 (0.09–6.4)	2.2 (0.13–7.9)	n.s
ΔSAT, BAR	-	0.065	0.063	n.s
ΔSAT, BAR%	-	3.08	3.12	n.s
PAT, BAR	0.16 (0.03–0.54)	0.16 (0.04–0.54)	0.18 (0.06–0.5)	n.s
ΔPAT, BAR	-	0.0039	0.0044	n.s
ΔPAT, BAR%	-	2.97	2.50	n.s
IMAT, BAR	0.54 (0.1–1.7)	0.49 (0.13–1.4)	0.57 (0.16–1.7)	< 0.05
ΔIMAT, BAR	-	–0.003	0.0045	< 0.05
ΔIMAT, BAR%	-	–0.62	0.60	< 0.05
EAT, BAR	0.08 (0.01–0.28)	0.08 (0.02–0.20)	0.08 (0.03–0.28)	n.s
ΔEAT, BAR	-	0.001	0.0043	n.s
ΔEAT, BAR%	-	1.2	4.38	n.s

*FEV*_*1*_ forced expiratory volume in 1 s, *MCID* minimally clinical important difference, *BAR* bone adjusted ratio, *IMAT* intra- and intermuscular adipose tissue, *EAT* epicardial adipose tissue, *PAT* paracardial adipose tissue, *SAT* subcutaneous adipose tissue, *TAT* total adipose tissue. *n.s*. non-significant. Statistically significant differences between groups tested with Mann–Whitney-test.

## Discussion

In this study, we assessed the impact of body composition on functional parameters before and after bronchoscopic lung volume reduction (BLVR) with valves. We utilized body composition analysis (BCA) through CT scans collected both before and after valve implantation. Our findings at baseline revealed moderate correlations between muscle volume and various outcome parameters, as well as weak negative correlations between adipose tissue volumes and specific outcomes. Throughout the follow-up period, we observed significant changes in adipose tissue volumes.

Our correlation analyses consistently demonstrated associations between muscle volume and outcome parameters at all evaluation points, with these associations becoming stronger over time, particularly at the 6-month follow-up. To explore the influence of muscle volume changes in greater depth, we conducted group differentiation based on these changes, revealing varying impacts on outcome parameters. While there was no statistically significant difference in the overall muscle volume adjusted for bone, there were individual variances that aligned with differences in outcome parameters. Notably, the group with the most substantial change in muscle volume exhibited more favorable outcomes.

Moreover, we conducted comparisons utilizing the MCID for FEV_1_ at the 6-month post-intervention period. This approach is utilized due to the fact that FEV_1_ can be actively improved through continuous training and physiotherapy, in contrast to RV, which cannot be actively influenced by the patients themselves. These analyses unveiled significant differences in bone-adjusted muscle and intermuscular adipose tissue (IMAT) volumes, altogether underscoring a close interplay of body composition, particularly bone adjusted muscle volume, and functional parameters, determined as clinical outcomes after bronchoscopic lung volume reduction in severe emphysema patients. Although it would have been desirable, baseline body composition features did not exhibit a predictive value prior to BLVR, as assessed in a multivariate linear regression analysis.

The impact of body composition on patients with COPD and emphysema has been extensively investigated, with various factors shown to influence pulmonary function and exercise capacity. Notably, the prevalence of sarcopenia, characterized by a decline in muscle volume, has been a focal point in these investigations. Recent meta-analyses conducted by Sepúlveda‐Loyola et al. and He et al. reported prevalence rates ranging from 15.5 to 34% in the examined studies. These findings suggest a higher prevalence of sarcopenia in patients at advanced disease stages and its association with poorer pulmonary function and exercise capacity^24,25^. Martínez‑Luna et al. demonstrated a strong association between low muscle mass and poor spirometry results, particularly in FEV_1_ and FVC^26^. More specifically, Park et al. discovered that a decrease in intercostal muscle mass and an increase in intercostal fat are linked to a similar decrease in FEV_1_ and an increase in emphysema, which generally contributes to the worsening of COPD severity^27^. Not only thoracic muscle mass and volume could be linked to pulmonary and physical function in COPD patients. Seymour et al. could show that quadriceps muscle weakness was associated with lower FEV_1_ and 6MWD values while Martinez et al. demonstrated that handgrip-strength significantly correlated with FEV_1_^14,28^.

Regarding the impact of adipose tissue, there appears to be a nonlinear relationship with its volume and disease severity. We demonstrated a notable overall rise in total adipose tissue following BLVR, although it did not exhibit a significant correlation with outcome parameters. While obesity is strongly linked to increased risks of morbidity, mortality, and compromised lung function, very low levels of adipose tissue also show a tendency to worsen pulmonary function parameters^29,30^. Shimada et al. discovered that both low fat and muscle mass contribute to emphysema severity, while reduced muscle mass alone is associated with a diminished quality of life. Additionally, Zagaceta et al. established a link between epicardial adipose tissue (EAT) volume, smoking history, BMI, and lower exercise capacity^31,32^. In our investigation we also found a weak but statistically significant negative correlation of EAT volume with the 6MWD at all times supporting the aforementioned evidence. Comparable to what was described earlier, we observed a correlation between decreased bone adjusted muscle volume, but not adipose tissue volume, and diminished QoL.

In patients who received lung volume reduction surgery (LVRS) a recovery of body composition and weight gain in nonobese patients came along with improved nutritional status, exercise capacity and pulmonary function parameters^33,34^. Sanders et al. assessed skeletal muscle and adipose tissue using a single-slice CT scan at the first lumbar level in post-BLVR patients. They observed a significant increase in muscle mass, SAT, and IMAT, along with an association between muscle remodeling and improved exercise capacity^35^. Similarly, we observed a significant elevation in IMAT levels among patients who met the MCID for FEV_1_.

In our current investigation, we provided further evidence supporting the previously mentioned insights. For the general study population there was a significant increase in the volume of bone adjusted total adipose tissue while muscle volume remained constant. Nevertheless, bone-adjusted muscle volume consistently exhibited the strongest correlations with functional parameters, both at baseline and after valve implantation. Notably, the positive relationship between muscle growth and favorable outcomes became increasingly pronounced, particularly at the 6-month post-intervention mark. This observation suggests that over a longer duration, the disparities in functional parameters between patients adhering to regular training regimens and those who do not become more active, emphasizing the crucial role of muscle involvement in the outcomes following BLVR. When comparing the clinical profiles among the four groups categorized by varying degrees of muscle growth, no significant differences were observed after multiple testing correction. However, a noteworthy distinction emerged in the adherence to regular training protocols, with a higher proportion of patients in the groups characterized by muscle growth actively engaging in training. Additionally, it's worth mentioning that the group with muscle growth exhibited the lowest rate of depression (Supplementary Table S3).

To evaluate the impact of the extent of volume reduction, we conducted separate group-differentiated tests for patients who achieved the MCID for RV and those who did not. Furthermore, we performed a multiple linear regression analysis a multiple linear regression analysis, adjusting for gender and age, to assess the combined impact of RV changes and muscle volume changes at the 6-month mark. For those who achieved the MCID for RV, we observed similar trends as mentioned earlier. However, in the group that did not achieve the MCID for RV, there was no statistically significant difference at the 3-month follow-up. Nonetheless, there was still a tendency towards favorable results for the group with higher changes in muscle volume. It's important to note that this observation should be interpreted cautiously due to the limited number of patients and numerical imbalances among the four groups. By the 6-month mark, the difference became statistically significant again. This suggests that the influence of bone adjusted muscle volume and its respective changes also hold significance for patients without a clinically significant volume reduction. The multivariate analysis showed that bone adjusted muscle volume contributed to significant improvements for all tested outcome parameters (FEV_1_, IVC, 6MWD and CAT) while RV showed no significant impact on 6MWD and CAT (Table 3).

In our research, we evaluated biometric information obtained from routine CT scans. Our novel approach aims to comprehensively quantify all body tissues from these scans, enhancing the practicality of BCA features in everyday clinical practice. Unlike many conventional BCA methods that are semi-automated or rely on reference regions, such as the lumbar vertebra (L3), to estimate subcutaneous adipose tissue, muscle, and bone volumes using a 2D U-Net architecture, our approach might offer a more refined and representative assessment. This is crucial because interindividual variability among patients is substantial, and relying solely on a single CT slice image at the L3 vertebra level may not accurately represent the entire body composition of a patient^36–38^. This becomes even more important in patients with severe emphysema, since there is a high variability in thoracic morphometry, lung configuration and emphysema^39^.

There are limitations to this study. First, this is a retrospective single-center investigation, therefore the results from our data must be confirmed in further, ideally prospective research. We identified alterations in body composition between baseline and follow-ups. Except for the mentioned connection between muscle gain and the higher proportion of training we could not identify the reasons for BCA alterations from the collected data. Furthermore, we quantified tissue volumes, which give no representation of functional capabilities. It is also worth mentioning, that group intervals were arbitrarily chosen, based on the intention to compare muscle loss versus gain, which would result in two groups. To provide a balanced representation of patients with varying degrees of gain or loss, we further divided the data into four groups.

Fully automated body composition analysis has been demonstrated to be an effective tool for the rapid and standardized assessment of bone, muscle and adipose tissue. BLVR enhances ventilatory mechanics, thereby improving the prerequisites for training, which, in turn, can lead to enhanced exercise capacity and pulmonary function. In terms of clinical implementation, it is crucial to emphasize the importance of adhering to training protocols both before and after valve implantation. Patients are frequently referred to pulmonary rehabilitation before BLVR to enhance exercise capacity. A similar emphasis on establishing and monitoring post-BLVR training protocols in the outpatient setting is necessary. Additionally, BCA analysis has the potential to serve as a valuable tool for detecting inadequate training progress or other contributing factors when unfavorable clinical outcomes are observed following lung volume reduction procedures.

## Conclusion

Our study underscores the significance of altered body composition, particularly the increase in muscle volume, as a contributing factor to functional improvements in patients who have undergone BLVR treatment. These findings highlight the potential role of body composition analysis in interpreting the outcomes of BLVR interventions.

### Supplementary Information

Supplementary Information.

## Data Availability

The datasets generated and analyzed during the current study are available from the corresponding authors on reasonable request.
