# Supplementary material for: Body composition impacts outcome of bronchoscopic lung volume reduction in patients with severe emphysema: a fully automated CT-based analysis
Source: Sci Rep. 2024 Apr 15;14:8718. doi: 10.1038/s41598-024-58628-0 (PMC11018765; doi:10.1038/s41598-024-58628-0)
Supplement: Supplementary file 1 — Supplementary Information. [file 41598_2024_58628_MOESM1_ESM.docx]

# **Supplementary Material**

Figure S1. Spearman correlation analysis of clinical outcome parameters at baseline, 3-month and 6-month follow-up with body composition at baseline, 3-month and 6-month follow-up. Values are correlation coefficients color-coded according to the level of significance (p<0.001, n.s.). 6MWD, 6-minute walking distance; CAT, COPD Assessment Test; FEV1, forced expiratory volume in 1 second; IVC, inspiratory vital capacity; RV, residual volume, BAR; bone adjusted ratio, IMAT, intra- and intermuscular adipose tissue; EAT, epicardial adipose tissue; PAT, paracardial adipose tissue; SAT, subcutaneous adipose tissue; TAT, total adipose tissue. We adjusted for multiple comparisons with Bonferroni correction.

Figure S2. Correlation analysis of clinical outcomes with body composition parameters. Correlation of changes in clinical outcome parameters with changes in bone adjusted muscle and total adipose tissue volume. Correlation coefficients are color-coded according to the level of significance (p<0.001, n.s.). 6MWD, 6-minute walking distance; CAT, COPD Assessment Test; FEV1, forced expiratory volume in 1 second; IVC, inspiratory vital capacity; RV, residual volume, BAR; bone adjusted ratio, IMAT, intra- and intermuscular adipose tissue; EAT, epicardial adipose tissue; PAT, paracardial adipose tissue; SAT, subcutaneous adipose tissue; TAT, total adipose tissue.

Table S1. Group differentiated analysis of outcome parameters according to changes in the bone adjusted ratio of muscle volume (RV≥MCID).

| Parameter | Pre-implant  (Baseline) | | | | |  | | Follow-Up  (3-Month) | | | | | | | |  | | Follow-Up  (6-Month) | | | | | | | | |  | | | |  |
| --- | --- | --- | --- | --- | --- | --- | --- | --- | --- | --- | --- | --- | --- | --- | --- | --- | --- | --- | --- | --- | --- | --- | --- | --- | --- | --- | --- | --- | --- | --- | --- |
|  |  | | | |  | |  | | | | | | | |  | |  | | | | | | | |  | | | |  |  |  |
| ΔMuscle,BAR % |  |  |  |  |  | | **<-10** | | **≤0** | | **>0** | | **>10** | |  | | **<-10** | | **≤0** | | **>0** | | **>10** | | |  | | | |  |  |
| *n* |  | 300 |  |  |  | | 29 | | 83 | | 78 | | 27 | |  | | 32 | | 48 | | 43 | | 21 | | |  | | | |  |  |
|  |  |  |  |  |  | |  | |  | |  | |  | | *p* | |  | |  | |  | |  | | | *p* | | | |  |  |
| FEV_1_, L |  | .75 |  |  |  | | .73 | | .86^*^ | | 1.0^*^ | | .9^*^ | | *<0.001* | | .65 | | .86^*^ | | .98^*^ | | .99^*^ | | | *<0.001* | | | |  |  |
| RV, L |  | 5.7 |  |  |  | | 4.8^*^ | | 4.7^*^ | | 4.8^*^ | | 4.4^*^ | | *n.s.* | | 4.7^**^ | | 4.4^*^ | | 4.8^*^ | | 4.0^*^ | | | *n.s.* | | | |  |  |
| IVC, L |  | 2.2 |  |  |  | | 2.4 | | 2.5^*^ | | 2.9^*^ | | 2.7^*^ | | *n.s* | | 2.3 | | 2.3 | | 3.1 | | 2.8 | | | *<0.001* | | | |  |  |
| 6MWD, m |  | 280 |  |  |  | | 265 | | 310^*^ | | 360^*^ | | 365^*^ | | *<0.001* | | 250^*^ | | 275 | | 402^*^ | | 420^*^ | | | *<0.001* | | | |  |  |
| CAT score |  | 27 |  |  |  | | 24^*^ | | 24^*^ | | 22^*^ | | 23^*^ | | *n.s.* | | 29^**^ | | 27 | | 22^*^ | | 22^*^ | | | *<0.001* | | | |  |  |
|  |  |  |  |  |  | |  | |  | |  | |  | |  | |  | |  | |  | |  | | |  | | | |  |  |
| ΔFEV_1_, L |  |  |  |  |  | | .08 | | .1 | | .15 | | .26 | | *<0.001* | | –.1 | | .06 | | .12 | | .32 | | | *<0.001* | | | |  |  |
| ΔFEV_1_, % |  |  |  |  |  | | 11 | | 14 | | 17 | | 41 | | *<0.001* | | –12 | | 8 | | 13 | | 53 | | | *<0.001* | | | |  |  |
| ΔRV, L |  |  |  |  |  | | –.9 | | –.9 | | –.8 | | –1 | | *n.s.* | | –1 | | –1 | | –1.1 | | –1.1 | | | *n.s.* | | | |  |  |
| ΔRV, % |  |  |  |  |  | | –15 | | –17 | | –15 | | –20 | | *n.s.* | | –18 | | –18 | | –18 | | –20 | | | *n.s.* | | | |  |  |
| ΔIVC, L |  |  |  |  |  | | .14 | | .25 | | .31 | | .56 | | *<0.001* | | –.22 | | .32 | | .34 | | .7 | | | *<0.001* | | | |  |  |
| ΔIVC, % |  |  |  |  |  | | 5.6 | | 12.5 | | 14 | | 28 | | *<0.001* | | –9 | | 12 | | 14 | | 29 | | | *<0.001* | | | |  |  |
| Δ6MWD, m |  |  |  |  |  | | 18 | | 27 | | 60 | | 120 | | *<0.001* | | –47 | | –10 | | 90 | | 145 | | | *<0.001* | | | |  |  |
| ΔCAT score |  |  |  |  |  | | –2 | | –2 | | –3 | | –3.5 | | *n.s.* | | 2 | | 1 | | –4 | | –6 | | | *<0.001* | | | |  |  |
|  |  |  |  |  | |  | |  | |  | |  | |  | |  | |  | |  | |  | |  | | | |  | | | |
|  |  | | | | |  | |  | | | | | | | |  | |  | | | | | | | | |  | | | |  |

6MWD, 6-minute walking distance; CAT, COPD Assessment Test; FEV_1_, forced expiratory volume in 1 second; IVC, inspiratory vital capacity; RV, residual volume, BAR; bone adjusted ratio, IMAT, intra- and intermuscular adipose tissue; EAT, epicardial adipose tissue; PAT, paracardial adipose tissue; SAT, subcutaneous adipose tissue; TAT, total adipose tissue. Values are displayed as median. Statistically significant differences between groups indicated with p-value and individual differences to baseline marked: *p<0.001. We adjusted for multiple comparisons with Bonferroni correction.

Table S2. Group differentiated analysis of outcome parameters according to changes in the bone adjusted ratio of muscle volume (RV<MCID).

| Parameter | Pre-implant  (Baseline) | | | |  | | Follow-Up  (3-Month) | | | | | | | |  | | Follow-Up  (6-Month) | | | | | | | |  | | |  |
| --- | --- | --- | --- | --- | --- | --- | --- | --- | --- | --- | --- | --- | --- | --- | --- | --- | --- | --- | --- | --- | --- | --- | --- | --- | --- | --- | --- | --- |
|  |  | | | |  |  | | | | | | | |  | |  | | | | | | | |  | | |  |  |
| ΔMuscle,BAR % |  |  |  |  |  | **<-10** | | **≤0** | | **>0** | | **>10** | |  | | **<-10** | | **≤0** | | **>0** | | **>10** | | |  | | |  |
| *n* |  | 300 |  |  |  | 4 | | 31 | | 18 | | 3 | |  | | 16 | | 24 | | 15 | | 11 | | |  | | |  |
|  |  |  |  |  |  |  | |  | |  | |  | | *p* | |  | |  | |  | |  | | | *p* | | |  |
| FEV_1_, L |  | .75 |  |  |  | .75 | | .7 | | .76 | | 1.13 | | *n.s.* | | .69^*^ | | .79^**^ | | .81^*^ | | .84^*^ | | | *<0.001* | | |  |
| RV, L |  | 5.7 |  |  |  | 5.7 | | 5.5^*^ | | 6^*^ | | 6.2 | | *n.s.* | | 5.1^*^ | | 5.5^*^ | | 5.4^*^ | | 5.0^*^ | | | *n.s.* | | |  |
| IVC, L |  | 2.2 |  |  |  | 2.1 | | 2.1 | | 2.2 | | 2.8 | | *n.s.* | | 2.1 | | 2.4^*^ | | 2.5^*^ | | 2.1^*^ | | | *<0.001* | | |  |
| 6MWD, m |  | 280 |  |  |  | 230 | | 240 | | 310 | | 430 | | *<0.001* | | 287^*^ | | 296 | | 303^*^ | | 320^*^ | | | *<0.001* | | |  |
| CAT score |  | 27 |  |  |  | 21 | | 22.5^*^ | | 22 | | 23 | | *n.s.* | | 27^*^ | | 25 | | 25 | | 22 | | | *<0.001* | | |  |
|  |  |  |  |  |  |  | |  | |  | |  | |  | |  | |  | |  | |  | | |  | | |  |
| ΔFEV_1_, L |  |  |  |  |  | .05 | | –.07 | | .06 | | .23 | | *n.s.* | | –.05 | | .04 | | –.03 | | .18 | | | *<0.001* | | |  |
| ΔFEV_1_, % |  |  |  |  |  | 5.7 | | –9 | | 6 | | 25 | | *n.s.* | | –5.5 | | 4.4 | | –1.8 | | 33 | | | *<0.001* | | |  |
| ΔRV, L |  |  |  |  |  | 0 | | .3 | | .25 | | .9 | | *n.s.* | | .35 | | .31 | | .31 | | .30 | | | *n.s.* | | |  |
| ΔRV, % |  |  |  |  |  | 0 | | 6.4 | | 4 | | 19 | | *n.s.* | | 7.1 | | 6.8 | | 6.8 | | 6.6 | | | *n.s.* | | |  |
| ΔIVC, L |  |  |  |  |  | .15 | | –0.03 | | –.09 | | .1 | | *n.s.* | | –.3 | | .01 | | .1 | | .4 | | | *<0.001* | | |  |
| ΔIVC, % |  |  |  |  |  | 5.5 | | –1.8 | | –4.4 | | 7.2 | | *n.s.* | | –11 | | 1.2 | | 6.4 | | 27 | | | *<0.001* | | |  |
| Δ6MWD, m |  |  |  |  |  | 5 | | –13 | | 12.5 | | 98 | | *n.s.* | | –4 | | 13 | | 16 | | 101 | | | *<0.001* | | |  |
| ΔCAT score |  |  |  |  |  | 0 | | –3 | | –1.5 | | –1 | | *n.s.* | | 2 | | 1 | | 2 | | –3 | | | *<0.001* | | |  |
|  |  |  |  |  |  | |  | |  | |  | |  | |  | |  | |  | |  | |  | | |  | | |
|  |  | | | |  | |  | | | | | | | |  | |  | | | | | | | |  | | |  |

6MWD, 6-minute walking distance; CAT, COPD Assessment Test; FEV_1_, forced expiratory volume in 1 second; IVC, inspiratory vital capacity; RV, residual volume, BAR; bone adjusted ratio, IMAT, intra- and intermuscular adipose tissue; EAT, epicardial adipose tissue; PAT, paracardial adipose tissue; SAT, subcutaneous adipose tissue; TAT, total adipose tissue. Values are displayed as median. Statistically significant differences between groups indicated with p-value and individual differences to baseline marked: *p<0.001. We adjusted for multiple comparisons with Bonferroni correction.

Table S3. Clinical profile and comorbidities (t=6 month).

| Parameter | |  | | | | |  | | |  |  | | |  | | p-value | |  |  |
| --- | --- | --- | --- | --- | --- | --- | --- | --- | --- | --- | --- | --- | --- | --- | --- | --- | --- | --- | --- |
|  | |  | | | | |  | | |  |  | | |  |  | |  |  |  |
| ΔMuscle,BAR % | | **<-10** | | **<0** | | **>0** | | | | | **>10** | | | |  | |  | | |
| *n* | | 45 | | 64 | | 62 | | | | | 29 | | | |  | |  | | |
|  |  |  |  |  |  |  |  |  |  |  |  |  |  |  |  |  |  |  |  |
| Age, years | | | 57 | | 58 | | | 60 | | | | | 58 | | | n.s. | |  |  |
| Gender, female | | | 27 (60) | | 45 (70) | | | 32 (52) | | | | | 32 (72) | | | n.s. | |  |  |
| Training p.i. | | | 16 (36) | | 29 (45) | | | 31 (50) | | | | | 20 (69) | | | n.s. | |  |  |
|  | | |  | |  | | |  | | | | |  | | |  | |  |  |
| CVD, n (%) | | | 11 (24) | | 16 (25) | | | 17 (27) | | | | | 6 (23) | | | n.s. | |  |  |
| Osteoporosis, n (%) | | | 7 (16) | | 14 (22) | | | 11 (18) | | | | | 4 (14) | | | n.s. | |  |  |
| Hyperlipidemia, n (%) | | | 6 (13) | | 14 (22) | | | 8 (13) | | | | | 5 (17) | | | n.s. | |  |  |
| CKD, n (%) | | | 1 (2) | | 4 (6) | | | 2 (3) | | | | | 1 (3) | | | n.s. | |  |  |
| DM II, n (%) | | | 2 (4) | | 4 (6) | | | 2 (3) | | | | | 2 (7) | | | n.s. | |  |  |
| Depression, n (%) | | | 8 (18) | | 8 (13) | | | 5 (8) | | | | | 2 (7) | | | n.s. | |  |  |
| Exacerbation p.i., n (%) | | | 4 (9) | | 5 (8) | | | 3 (5) | | | | | 2 (7) | | | n.s. | |  |  |
| Pneumothorax, n (%) | | | 11 (24) | | 8 (13) | | | 12 (19) | | | | | 5 (17) | | | n.s. | |  |  |
|  | |  | | | | | | |  | | |  | |  |  | |  | |  |

Values are number of patients and percentage (%).

BAR; bone adjusted ratio, n.s.; non-significant, CKD; chronic kidney disease, CVD; cardio-vascular disease (arterial hypertension, peripheral artery disease, coronary heart disease), DM II; diabetes mellitus type II. p.i.; post-intervention. We adjusted for multiple comparisons with Bonferroni correction.
